# Tri-Modal Cytology Excellence: Improving Body Fluid Diagnostic Performance

**DOI:** 10.22034/ijp.2026.2082834.3618

**Published:** 2026-08-01

**Authors:** Divya M, Margaret Theresa, Birundha Baskaran, Prem kumar, Jeya Shambavi, Jawahar Ramasamy

**Affiliations:** 1 Department of Pathology, Aarupadai Veedu Medical College and Hospital, Vinayaka Mission’s Research Foundation (Deemed to be university), Puducherry, India; 2 Department of General Medicine, Aarupadai Veedu Medical College and Hospital, Vinayaka Mission’s Research Foundation (Deemed to be university), Puducherry, India

**Keywords:** Cell block, cytospin, conventional smear, effusion cytology, body fluids

## Abstract

**Background & Objective:**

Cytological examination of effusion fluids is a valuable diagnostic tool. In India, conventional smear preparations are widely used, but they are often limited by poor cellularity, overlapping cells, and difficulty distinguishing malignant cells from reactive mesothelial cells. Cytospin and cell block techniques overcome these limitations by improving cellular distribution, preservation, and morphology. This study evaluates the merits and demerits of conventional smear, cell block, and cytospin methods in effusion cytology.

**Methods:**

A cross-sectional study was conducted over 18 months in the Department of Pathology in a tertiary health care center. A total of 170 body fluids and urine samples were processed by conventional smear, cell block, and cytospin techniques for comparison. IHC was done for selected malignancy cases in cell block preparation.

**Results:**

Cytospin (51%) and cell block (46%) yielded superior background clarity compared with conventional smear (3%). Cellular yield was better with cell block (62%) and cytospin (36%) than conventional smear (2%). Morphology of cells was better preserved in cytospin (54%) than cell block (45%) and conventional smear (1%). Distribution of cells seemed to be better in cytospin (52%) than cell block (43%) and conventional smear (5%).

**Conclusion:**

Cytospin provides better morphology, background, and distribution of cells compared with conventional smears. Cell block excels in cellularity, while conventional smears remain limited by poor quality with low cellularity, high degeneration, and poor distribution of cells, making cell block and cytospin more effective for effusion cytology.

## Introduction

Effusion refers to the accumulation of fluid within a body cavity, such as the peritoneal cavity, pleural cavity, or pericardial cavity, and arises due to various medical conditions, including infection, inflammation, trauma, systemic diseases, and malignancies(1). Cytological evaluation of these effusions is crucial for establishing the underlying cause and guiding treatment(2,3). Malignant effusions often indicate advanced disease and carry significant prognostic implications(4,5).

The conventional smear technique is a widely used method in India due to its simplicity and affordability(6). However, this is often limited by poor cellular yield, background obscuration by blood or proteinaceous material, and difficulty in distinguishing reactive mesothelial cells from malignant cells. This can lead to inconclusive reports, delay in final diagnosis, and appropriate management(7,8).

To overcome this, other cytological preparation techniques, such as cell block and cytospin, have been developed(9,10). The cell block technique allows concentration of cells into a paraffin-embedded block, enabling better cellularity, architectural preservation, and application of ancillary tests like immunohistochemistry(11). Cytospin concentrates cells onto a small area of a slide, producing thin-layer preparations with better background clarity and enhanced visualization of cellular details(12).

Few studies have evaluated the advantages of cell block and cytospin over conventional smears. Since no single technique has been universally adopted for the diagnosis of effusions, the present study aims to compare the diagnostic yield of conventional smears, cell block, and cytospin techniques in body fluid cytology using objective scoring parameters, with the goal of identifying the most effective approach to enhance diagnostic accuracy.

## Materials and Methods

### Study Design and Population

This is a cross-sectional study conducted in the Department of Pathology, in a Medical College Hospital, Puducherry, over a period of 18 months. Institutional ethical clearance and informed consent were obtained from all patients prior to sample collection. A total of 160 body fluid samples were studied. Sample size of 160 was calculated as per estimation of sensitivity of cytospin (0.818) and absolute precision of 6% with the reference of Mashru et al(14).

### Inclusion and Exclusion Criteria

All types of body fluids from patients belonging to all age groups (pleural, peritoneal, pericardial, bronchial brush cytology, bronchoalveolar lavage, synovial fluid, urine samples) meeting the study’s minimal volume requirement for proper processing (>20 mL), CSF (>2 mL), and transported within 24 hours of collection at appropriate temperature as per standard operating protocol. Samples below the minimal volume for processing, clotted samples, improperly stored samples, hemorrhagic samples, and contaminated samples were excluded from the study to ensure uniform preparation across techniques. While this ensured technical comparability, it may introduce selection bias and limit applicability in low-volume or suboptimal samples encountered in routine practice. Future studies may specifically evaluate technique performance in hemorrhagic and low-quality samples.

### Processing Techniques and Scoring

Each fluid sample was divided into three aliquots and processed using three different cytological techniques. From one part of the sample, conventional smears were prepared from centrifuged deposits, air-dried, and stained with hematoxylin and eosin and Papanicolaou stains as per standardised protocol. The second part of the sample was allowed to sediment and was fixed in formalin, processed, and embedded in paraffin. Sections were cut at 4-5 μm and stained with hematoxylin and eosin as per standardised protocol. A 0.5- to 1-mL aliquot of the third part of the sample was loaded in cytospin and centrifuged at 800 rpm for 10 minutes. All the body fluid samples were processed using standardised institutional protocols to ensure methodological consistency. The deposit obtained onto the glass slides was stained and scored under four criteria. Slides were scored using Sudha et al.(13) scoring system under four categories:

### Background: Obscuring blood/proteinaceous material (0-2 points).

Cellularity: Amount of diagnostic cells (0-2 points).

Morphology: Preservation vs degeneration of cells (0-2 points).

Distribution: Evenness of cell spread (0-2 points).

### Statistical Analysis

This was a cross-sectional analytical study designed to compare the morphological and technical parameters across three different cytological preparation techniques at a single point of time. It is more of a methodological comparison rather than a longitudinal diagnostic outcome assessment. Slides were independently evaluated by two experienced cytopathologists blinded to each other’s interpretation and preparation technique. This helped us to eliminate observer-related variability in interpretation and scoring. Data were analyzed using SPSS version 29. The results were expressed as mean ± standard deviation. Differences between techniques were analyzed using one-way ANOVA. P < .05 was considered statistically significant.

## Results

### Demographic and Clinical Profile

Among 170 fluid samples, peritoneal fluid was the predominant fluid, and the least predominant fluid was CSF (Table 1). The majority of the sample population, 158 (92%), were between 21 and 60 years, with peak incidence at 41 to 60 years, 83 (48%). Most of the population was male, with a predominance of 101 cases (59%)

**Table 1:** Distribution of different body fluids

Type of Fluid	N	Percentage
Peritoneal	74	43.5%
Pleural	58	34.2%
Synovial	25	14.7%
Pericardial	5	2.9%
Urine	5	2.9%
CSF	3	1.8%

Most of the cases were found to be of benign etiology, 142 cases (83.5%), and malignant cases were about 28 (16.5%). Peritoneal fluid, 14 (8%), and pleural fluid, 11 (6%), accounted for most of the malignant cases.

### Comparative Performance of Cytological Techniques

**Fig. 1:**
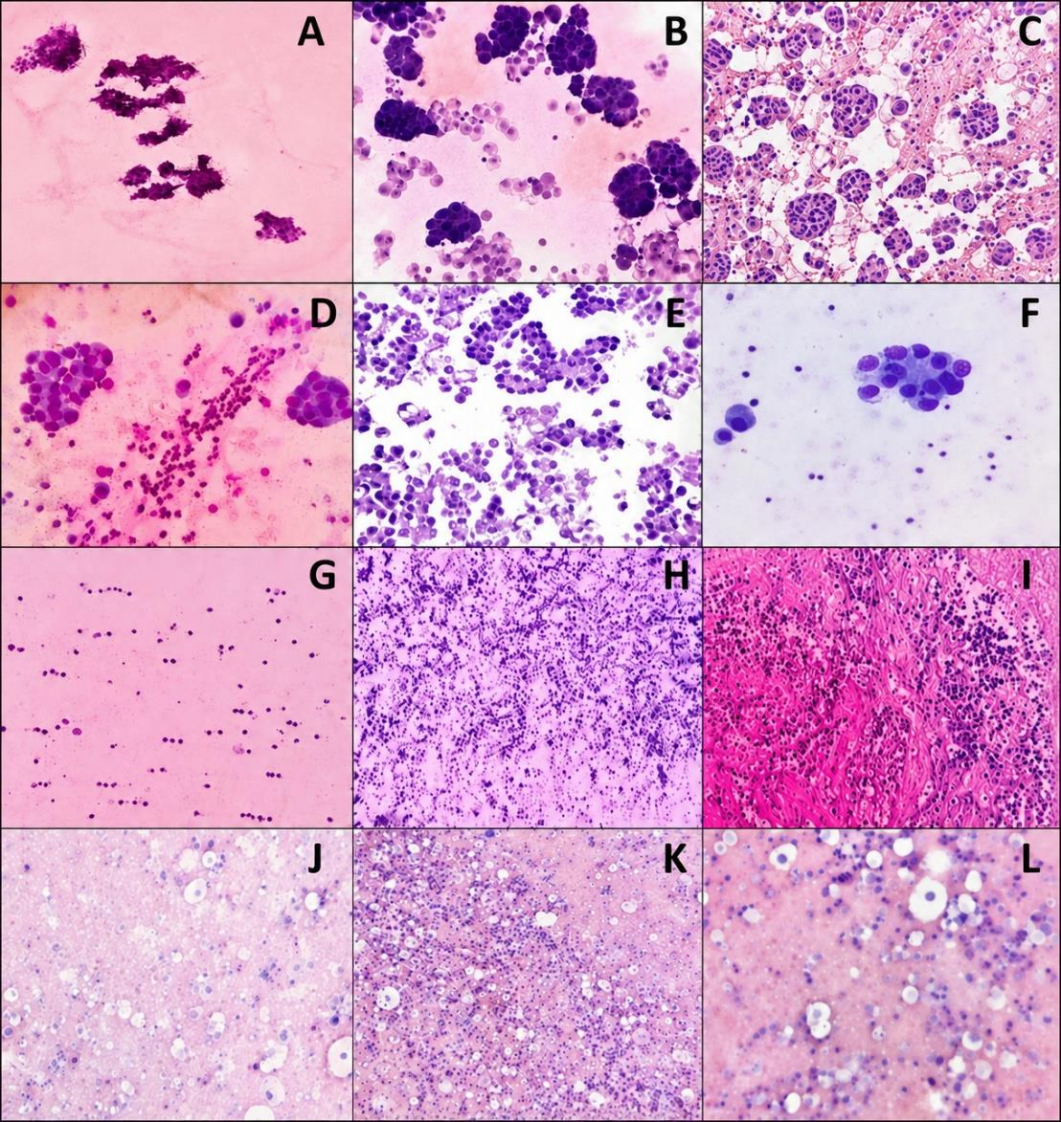
(A) depicts the conventional smear image positive for malignancy in pleural fluid cytology with atypical cells in a proteinaceous background (H&E, 10×). (B) shows cell block preparation of pleural fluid showing a cellular smear with atypical mesothelial cells (H&E, 40×). (C) shows cytospin smear preparation of pleural fluid malignancy in which the tumor cells are arranged in a glandular pattern, raising the suspicion of adenocarcinoma. IHC should be done to rule out adenocarcinoma and mesothelioma. (D) shows conventional smear study in pericardial fluid positive for malignancy in an inflammatory background (H&E, 40×). € is the cell block image of pericardial fluid with high cellularity and glandular arrangement of tumor cells (H&E, 40×). (F) shows the cytospin image of pericardial fluid with atypical cells exhibiting scant cytoplasm and hyperchromatic nuclei (H&E, 40×). (G) is the conventional smear image of lymphocytic effusion in peritoneal fluid (H&E, 10×). (H) is the cell block preparation of lymphocytic effusion in peritoneal fluid (H&E, 10×). (I) shows the cytospin smear (H&E, 10×). (J) shows the conventional smear study of high-grade urothelial carcinoma in urine. (K) is the cell block preparation of high-grade urothelial carcinoma in urine. Picture L is the cytospin method done in urine of high-grade urothelial carcinoma with a high N:C ratio and hyperchromatic nuclei.

On scoring, cytospin and cell block procedures produced substantially clearer backgrounds. Conventional techniques resulted in poorer background quality, limiting accurate diagnosis. The cell block approach outperformed other procedures in terms of cellularity. The conventional approach was the least effective, with most samples being nondiagnostic due to low cellularity. Cytospin and cell block procedures were preferable in terms of preservation of cell morphology, which improves cytological diagnostic accuracy. The conventional approach caused high levels of cellular degeneration, rendering a large portion of samples nondiagnostic or suboptimal. The cytospin and cell block methods were markedly superior in terms of cellular distribution. The conventional smear approach led to cell clustering at the periphery or sparse fields, which made the diagnosis less reliable (Table 2). Cell distribution, morphology, and background of body fluids processed by three different techniques are depicted in Fig 1.

**Table 2:** Comparative scoring of each technique

Parameter	Quality/Grade	Conventional Smear (%)	Cell Block (%)	Cytospin (%)	p-Value	Key Findings
Background Clarity	Poor	84 (72.4)	80 (43.2)	6 (2.9)	<0.001	Cytospin shows clear background
	Moderate	1 (0.9)	73 (39.5)	96 (46.2)		
	Clear	32 (26.7)	32 (17.3)	106 (51)		
Cellularity	Poor	109 (64.5)	1 (0.6)	60 (34.7)	<0.001	Cell block yields max cellularity
	Moderate	55 (33.7)	65 (39.2)	50 (30.6)		
	Abundant	4 (1.8)	106 (60.2)	60 (34.7)		
Morphology Preservation	Degeneration	59 (62.1)	108 (52)	3 (1)	<0.001	Cytospin provides better detail
	Moderate	25 (26.3)	53 (25)	92 (45)		
	Minimal	11 (11.6)	48 (23)	111 (54)		
Cell Distribution	Poor	63 (74)	98 (42)	9 (5)	<0.001	Cytospin best for uniform spread
	Intermediate	19 (23)	68 (29)	83 (43)		
	Even	3 (3)	68 (29)	99 (52)		

### Role of Ancillary Testing

IHC performed on cell blocks acted as an adjunct to the cytomorphological diagnosis and for definitive subtyping of malignant lesions. By analyzing the cytomorphological features, we can identify the origin of malignancy in a metastatic site.

**Fig. 2:**
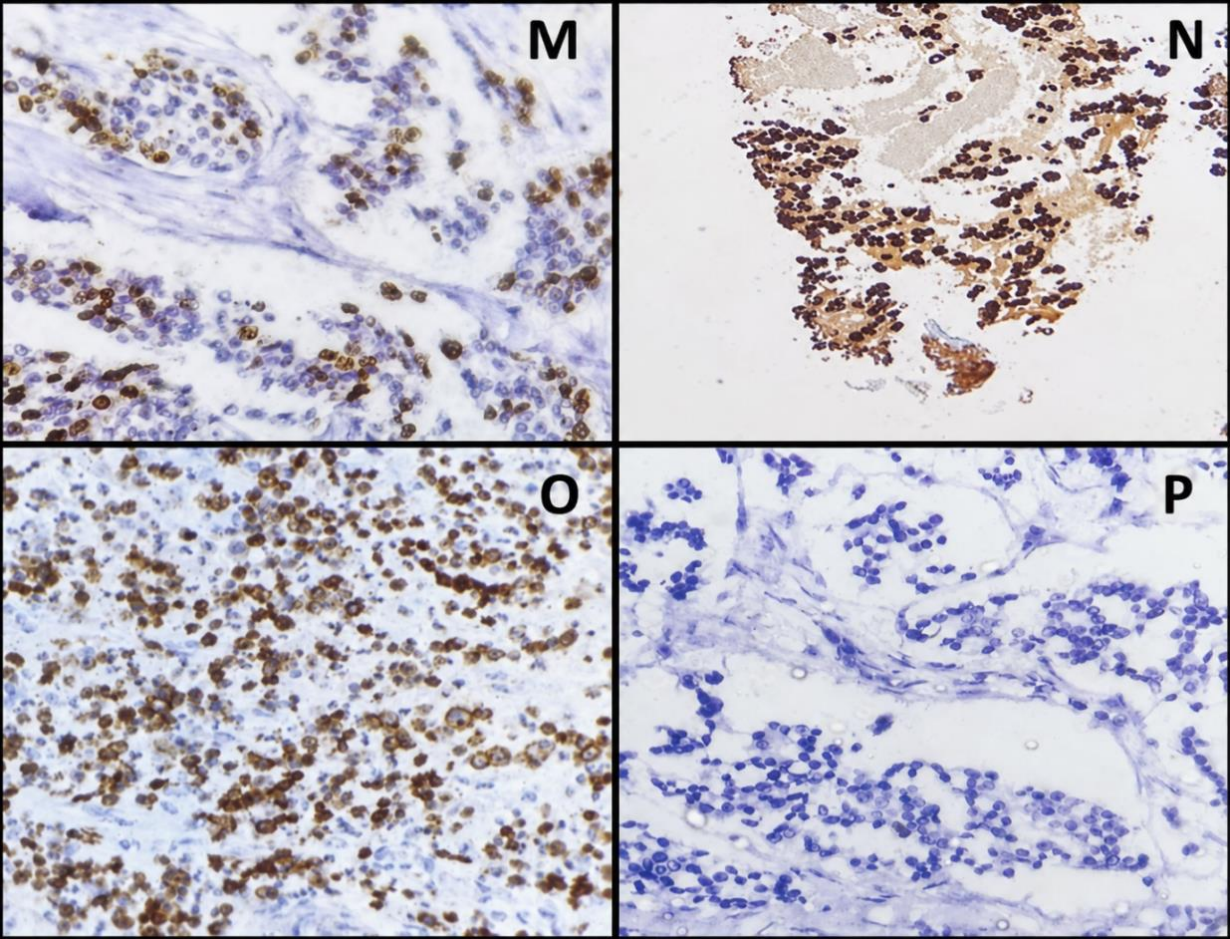
Immunohistochemistry on cell blocks. (M) Ki-67 positivity in >30% of cells, malignant pleural effusion (×40). (N) PAX-8 nuclear positivity, high-grade urothelial carcinoma in urine (×40). (O) CK7 positive and (P) CK20 negative in tumor cells of peritoneal fluid, consistent with gastric epithelial origin (×40).

In our study, for cases identified with malignant effusions, we confirmed the diagnosis with immunohistochemistry. Ki-67 was done on malignant effusion diagnosed in pleural fluid and was found to be positive in >30% of cells (Fig 2M).

PAX-8 nuclear positivity was found in high-grade urothelial carcinoma deposits in urine (Fig 2N).

CK7 and CK20 were done on peritoneal fluid cell block, and it was found that CK7 was positive and CK20 was negative in the tumor cells of peritoneal fluid, giving us a clue that the origin of malignancy was from gastric epithelium (Fig 2O, 2P).

## Discussion

Serous effusion cytology is the initial diagnostic step in determining the etiology(15,16). The majority of laboratories use the conventional smear technique, although methods vary in different laboratory setups(17). In conventional smear cytology, differentiating reactive mesothelial cells from neoplasm is one of the most frequent issues(18,19). Cytological examination is very helpful in the therapy of malignant neoplasms, especially lymphoid neoplasms, which are a major cause of death in children(20,21). In cases of malignancy, the cell block technique plays a major role, as immunohistochemistry acts as an added advantage(22,23). Cytospin is particularly useful in samples with low cellularity. It preserves cell morphology better(24,25). The major drawback of cell block is its longer turnaround time resulting from overnight tissue processing, which could delay diagnosis(26,27). The cytological examination of serous effusions has increasingly gained acceptance in clinical medicine(28). The results of several independent effusion cytology investigations showed that the cytospin and cell block techniques are superior to the conventional method for diagnosing effusions(29,30). It is crucial not only for the diagnosis of malignant lesions but also for staging and prognosis (. According to various studies, if conventional smear is supplemented by cell block and cytospin techniques, additional diagnostic yield for malignancy is noted(29,30).

The present study compares three cytological techniques in evaluating body fluids. Our results demonstrate that cell block and cytospin significantly outperform conventional smears among all diagnostic parameters. Although this was a single-center study, uniform processing protocols and inclusion of multiple body fluid types enhance internal validity. Multicentric studies with larger cohorts are recommended to validate these findings across diverse laboratory settings.

Background clarity: Conventional smears frequently showed obscuring proteinaceous debris and blood, reducing diagnostic accuracy. Cytospin, which concentrated cells into a limited area, produced a clearer background, consistent with Deshpande et al findings(12).

Cellularity: Cell block has the maximum cellular yield, confirming findings with Kumar et al and Mashru et al(26,14). The capacity of cell blocks to capture and concentrate diagnostic cells into paraffin blocks enables the later application of immunohistochemistry, which improves diagnostic precision in malignant effusion.

Morphology: Both cell block and cytospin preserved morphology better than conventional smears, with cytospin performing slightly better. This finding is consistent with research by Matreja et al and Batool et al, who emphasized the importance of cell block in architectural detail and cytospin in nuclear morphology(23,9).

Distribution: Cytospin provided the most uniform cell spread, making screening easier and lowering the risk of overlooking malignant cells. This confirms the findings of Joseph et al, who noted superior cell distribution in cytospin preparations(27).

## Clinical Implications

The high frequency of benign effusions in our study (83%) is consistent with global literature, but the malignancy (16%) discovery emphasizes the importance of cytology in the early detection of metastatic disease. Adopting combined cytospin and cell block approaches in everyday practice can reduce false negatives and maximize diagnostic confidence.

Cytological investigation of body fluids remains a useful first-line diagnostic tool. Our results show that cell block and cytospin outperform conventional smears in terms of background, cellularity, morphology, and distribution. Cell block excels at providing abundant cellularity and preserved architecture, whereas cytospin ensures optimal background and distribution.

Although multiple fluid types were included in the study to increase applicability, variation in intrinsic cellularity may have influenced comparative performance. As this study focused primarily on technical comparison between preparation methods, diagnostic accuracy could not be assessed due to lack of uniform histopathological or clinical follow-up. Further prospective longitudinal studies with follow-up data are warranted to evaluate the impact of preparation technique on clinical outcomes and diagnostic concordance over time.

## Limitations

This cross-sectional, single-center study with a limited sample size may restrict generalizability of the findings. Lack of uniform histopathological or clinical follow-up prevented the assessment of diagnostic accuracy. Observer-related subjectivity and inclusion of only adequate samples may influence reproducibility and real-world applicability of the results.

## Conclusion

We recommend the combined use of cell block and cytospin techniques with conventional smears in effusion cytology to improve diagnostic yield, facilitate ancillary testing, and reduce false negatives. This integrated approach can significantly enhance patient care, especially in distinguishing malignant from benign effusions. Each method has unique strengths, and our findings highlight that no single technique is sufficient across all sample types. A trimodal approach ensures an increase in overall diagnostic accuracy. Integrating all three methods into routine practice, especially in low-volume or low-cellularity samples, elevates the quality and reliability of body fluid cytology diagnostics.

## Data Availability

The data that support the findings of this study are available from the corresponding author upon request.
